# Discovery of a High 3-Hydroxyhexanoate Containing Poly-3-hydroxybutyrate-*co*-3-hydroxyhexanoate Producer-, *Cupriavidus* sp. Oh_1 with Enhanced Fatty Acid Metabolism

**DOI:** 10.3390/polym17131824

**Published:** 2025-06-30

**Authors:** Gaeun Lim, Suk-Jin Oh, Yebin Han, Jeonghee Yun, Jeong Chan Joo, Hee-Taek Kim, Hyun Gi Koh, See-Hyoung Park, Kyungmoon Park, Yung-Hun Yang

**Affiliations:** 1 Department of Biological Engineering, College of Engineering, Konkuk University, Seoul 05029, Republic of Korea; lge0919@naver.com (G.L.); equal73@naver.com (S.-J.O.); hanyebin3@gmail.com (Y.H.); 2 Department of Forest Products and Biotechnology, Kookmin University, Seoul 02707, Republic of Korea; yunjh@kookmin.ac.kr; 3 Department of Chemical Engineering, Kyung Hee University, Yongin-si 17104, Republic of Korea; jcjoo@khu.ac.kr; 4 Department of Food Science and Technology, Chungnam National University, Daejeon 34134, Republic of Korea; heetaek@cnu.ac.kr; 5 Department of Biological and Chemical Engineering, Hongik University, Sejong 30016, Republic of Korea; hgkoh@hongik.ac.kr (H.G.K.); shpark74@hongik.ac.kr (S.-H.P.); pkm2510@hongik.ac.kr (K.P.); 6 Institute for Ubiquitous Information Technology and Applications, Konkuk University, Seoul 05029, Republic of Korea

**Keywords:** Polyhydroxyalkanoates, 3-hydroxyhexanoate, soybean oil, *Cupriavidus necator*

## Abstract

Poly(3-hydroxybutyrate-co-3-hydroxyhexanoate) (P(3HB-*co*-3HHx)) is a representative PHA copolymer that can improve the mechanical limitations of polyhydroxybutyrate (P(3HB)). Although genetic engineering can facilitate 3HHx incorporation, it often compromises cell growth and reduces polymer molecular weight owing to metabolic disruptions caused by the deletion of acetoacetyl coenzyme A (acetyl-CoA) reductase (PhaB). To address this issue, native strains capable of producing high levels of 3HHx were identified via oil-based *Cupriavidus* screening. Eight PHA-producing strains were isolated from various samples and *Cupriavidus* sp. Oh_1 exhibited the highest polyhydroxybutyrate (PHB) production at 15.23 g/L from 17.2 g/L of biomass using soybean oil. Moreover, Oh_1/*phaC*_Ra_*J*_Pa_, containing enoyl-CoA hydratase (*phaJ*) and PHA synthetase (*phaC*), was identified as the most effective novel strain producing the highest 3HHx mole fraction, 48.93 g/L of PHA from 52.3 g/L of biomass and achieving a maximum 3HHx accumulation of 27.2 mol%. The resulting P(3HB-*co*-3HHx) showed a higher Mw (12.3 × 10^5^) compared with P(3HB-*co*-3HHx) produced by the *phaB*-deleted strain (14.6 × 10^4^). Higher production of 3HHx was attributed to the higher expression of *phaC*_Ra_ and *phaJ*_Pa_ in Oh_1, with log2 fold changes of 2.94 and 8.2, respectively, as well as the upregulation of certain β-oxidation encoding operons. Collectively, these findings highlight a strain capable of synthesizing a substantial 3HHx fraction without requiring gene deletions or extensive genetic modifications.

## 1. Introduction

Polyhydroxyalkanoates (PHAs) are sustainable biopolymers that can be used as alternatives to fossil fuel-based plastics because of their biodegradability and biocompatibility. They can be synthesized from renewable carbon sources and exhibit diverse chemical properties [1]. PHAs are divided into short-chain-length PHAs (scl-PHAs; C_3_–C_5_), medium-chain-length PHAs (mcl-PHAs; C_6_–C_14_), and long-chain-length PHAs (lcl-PHAs; C_15_ or more), depending on the number of carbon atoms in their monomers [2]. Among these, poly(3-hydroxybutyrate-*co*-3-hydroxyhexanoate) [P(3HB-*co*-3HHx)] copolymers are remarkable PHAs for practical applications compared with P(3HB) homopolymers because of their advanced flexibility and compatibility with the properties of petroleum-based plastics [3]. *Aeromonas caviae* was identified as the first organism capable of synthesizing P(3HB-*co*-3HHx); however, the amount of PHA produced was low [4].

*Cupridavidus necator* is one of the most renowned bacteria that can utilize various carbon substrates for polyhydroxybutyrate (PHB) production; it exhibited rapid growth and achieved high cell densities [5]. However, the PHA synthase of wild-type *C. necator* inherently exhibited a higher affinity for short-chain length monomers than for medium-chain length monomers, resulting in a strong preference for SCL-PHA production [6].

Subsequent investigation into the incorporation of the PHA synthase gene into *C. necator* H16 enabled the synthesis of P(3HB-*co*-3HHx); however, a low 3HHx mole fraction was observed in the synthesized copolymers [7,8]. To address these limitations, two approaches have been suggested to enhance 3HHx incorporation (Table 1). First, deleting native *phaC* and introducing external *phaC* from another species has been shown to increase 3HHx synthesis in P(3HB-*co*-3HHx) copolymers [9]. For instance, substituting *C. necator*’s native *phaC* with that from *Rhodococcus aetherivorans* I24 resulted in enhanced 3HHx incorporation when grown in plant oils [10]. The second strategy involved the deletion of *phaB* and the introduction of enoyl-CoA hydratase (*phaJ*) [11]. The deletion of *phaB* reduced the supply of HB-CoA monomers and redirected monomer flux to enhance 3HHx incorporation [12]. Although *phaJ* encoded one of the key enzymes responsible for supplying (R)-3HHx-CoA via *β*-oxidation, enzymes encoded by the endogenous *phaJ* in *C. necator* exhibit low affinity for MCL-PHA, and different *phaJ* variants exhibited distinct substrate specificity. Therefore, introducing *phaJ* variants with a higher affinity for MCL monomers could further improve 3HHx incorporation [13].

These genetic engineering approaches could effectively enhance P(3HB-*co*-3HHx) production, along with an increase in the 3HHx mole fraction of copolymers; however, the deletion of endogenous PHA synthase might adversely affect the overall metabolism of *C. necator*. For example, deleting *phaB* could disrupt the supply of HB-CoA and the intracellular redox balance by altering NADPH consumption. This metabolic perturbation downregulated the expression of genes involved in carbon and energy metabolism, resulting in reduced cell growth, limited carbon utilization, and decreased PHA production [14,15]. Furthermore, the Δ*phaB* mutant strain produced PHA with reduced molecular weight and increased polydispersity owing to the intracellular degradation and conversion of the stored polymer [10]. Given these challenges, different strategies were still needed for further applications [16].

Here, we investigated strains with the innate capacity to synthesize a high mole fraction of 3HHx from fatty acids without deleting the PHA synthesis gene. To achieve this, an oil-based screening was performed, and eight strains were isolated from various sources. *Cupriavidus* sp. Oh_1 exhibited the highest PHB yield at various concentrations of soybean oil. P(3HB-*co*-3HHx), with a high 3HHx mole fraction, was synthesized from soybean oil using Oh_1 harboring *phaC* from *R. aetherivorans* and *phaJ* from *Pseudomonas aeruginosa*. Considering that many studies have focused on *C. necator* H16-based genetically engineered strains, identifying a novel strain that exhibits a superior inherent ability to synthesize 3HHx represents a promising alternative strategy to control 3HHx content in polymer synthesis without gene deletion.

**Table 1:** Strategies to increase the 3-hydroxyhexanoate incorporation in *C. necator* from various fatty acids.

Strategies	Strains	Genetic Construct	Fatty Acids	DCW (g/L)	3HHx (mol%)	Ref.
Introduction of *phaC*	H16dphaC1	*phaC_Ac_*	Octanoate	2.6	2	[17]
			Bean	4.1	11	
	H16dphaC1	*phaC_Ac_*	Palm kernel	165	8	[18]
		*phaC_Ac_* + *BktB*	Palm kernel + butyrate	170	13	
Δ*phaC* + *phaC*	*C. necator* DMS 541	*phaC1_Re_*, *phaB_Re_*	Hexanoate	1.9	10	[19]
	*C. necator* DMS 541	*phaC_Ac_ NSDG*	Soybean	2.3 ± 0.2	5.2 ± 0.3	[7]
	Re2000	Δ*phaC1* + *phaC1_Ra_*	Palm	7.3 ± 0.1	1.1 ± 0.3	[10]
	Re2001	Δ*phaC2* + *phaC1_Ra_*	Palm	2.2 ± 0.1	1.5 ± 0.2	
	Re1034/pCB81	Δ*phaC1* + (*phaC_Ra_* + *phaA_Cn_* + *phaJ_Pa_*)	Palm	4.0 ± 0.2	11.6 ± 0.2	
	Re2058/pCB113	Δ*phaC1*, Δ*proC* + (*phaC_Ra_* + *phaA_Cn_* + *phaJ_Pa_* + *proC_Cn_*)	Palm	3.6 ± 0.3	12.7 ± 0.3	
	Re2135	Δ*phaC1*, Δ*phaB1*, *2*, *3* + *phaC2_Ra_*	Palm	1.22 ± 0.08	31.4 ± 0.8	
Δ*phaB,C* + *phaJ* and/or *phaC*	Re2133/pCB81	Δ*phaC1*, Δ*phaB1*, *2*, *3* + (*phaC_Ra_* + *phaA_Cn_* + *phaJ_Pa_*)	Palm	2.9 ± 0.1	23.3 ± 0.2	
	Re2152	Δ*phaC1*, Δ*phaB1*, *2*, *3* + *phaC2_Ra_* + *phaJ1_Pa_*	Palm	1.40 ± 0.02	22.4 ± 0.1	
	NSDGΔAC	Δ*phaC*, *phaA* + (*phaC_NSDG_* + *phaJ_Ac_*)	Bean	4.8 ± 0.05	9.9 ± 0.1	[20]
		Δ*phaC_Re_*, *phaA*, *fadB1* + (*phaC_NSDG_* + *phaJ_Ac_* + *phaJ4a_Re_*)	Bean	4.7 ± 0.1	11.7 ± 0.8	[21]
New strain	*Oh_1*	*phaC_Ra_ + phaR_Pa_*	Bean	16.4	24.3	This study

**Abbreviations:** DCW, dry cell weight; HHx, hydroxyhexanoate.

## 2. Materials and Methods

### 2.1. Isolation and Screening of Strain

Four samples were obtained from distinct locations: lacustrine sediment from Seoul Children’s Grand Park, mountain soil located behind the Moegaram Pension and Ganghwa Island, and freshwater from the Gongjicheon. An appropriate amount of soil samples was transferred into conical tubes, and sterilized distilled water was subsequently added. Following homogenization, the mixtures were left to settle, after which the supernatant was collected and diluted 10-fold with sterile distilled water. The liquid samples (freshwater) were directly diluted 10-fold. Then, 100 µL of the diluted sample was spread onto tryptic soy agar (TSA) plate containing gentamycin (Gm) and incubated at 30 °C for 48 h. After incubation, colonies were selected and re-streaked onto fresh TSA-Gm plates. To evaluate PHB production, the transferred colonies were initially grown in tryptic soy broth (TSB) supplemented with Gm and then incubated for 72 h in *Ralstonia eutropha* minimal medium (ReMM) supplemented with 1% fructose and 0.1% urea. PHA was quantified using GC-FID to identify the PHB-producing strains.

### 2.2. 16s rRNA Sequencing

All isolated strains were identified by 16S rRNA sequencing and PCR amplification using the 27F primer. Partial sequences were obtained from Bionics Inc. (Seoul, Korea) and compared with those in the National Center for Biotechnology Information GenBank database using BLAST (https://blast.ncbi.nlm.nih.gov/Blast.cgi, accessed on 12 September 2024) [22]. BLAST analysis showed 99.16% sequence similarity between Oh_1 and *Cupriavidus necator* H16 E6A55_RS08365. This partial sequence of *Cupriavidus* sp. Oh_1 was used to construct a phylogenetic tree using MEGA X software (version 11.0.13, MegaSoft, LCC., Tempe, AZ, USA).

### 2.3. Culture Conditions for PHA Synthesis

Seed cultures of the PHB-producing strains and pBBR1MCS2::*phaC*_Ra_::*phaJ_Pa_* vector-harboring strains were cultured at 30 °C for 1 day in TSB medium (5 mL volume) supplemented with 10 µg/mL gentamycin and 300 µg/mL kanamycin. The pBBR1MCS2::*phaC*_Ra_::*phaJ_Pa_* vector, originally maintained in *Escherichia coli* DH5α, was sourced from our laboratory plasmid library. A 5× ReMM (20 g/L NaH_2_PO_4_, 23 g/L Na_2_HPO_4_, 2.25 g/L K_2_SO_4_), 100× MgSO_4_ (39 g/L MgSO_4_), 100× CaCl_2_ (6.2 g/L CaCl_2_), and 1000× trace elements (15 g/L FeSO_4_•7H_2_O, 2.4 g/L MnSO_4_•H_2_O, 2.4 g/L ZnSO_4_•7H_2_O, and 0.48 g/L CuSO_4_•5H_2_O dissolved in 0.1 M hydrochloric acid) solution was used for PHA production. These ‘×’ designations refer to stock solutions, which were added to the culture medium to achieve final culture concentration of 1×. The 5× ReMM solution was sterilized by autoclaving and the 100× MgSO_4_, 100× CaCl_2_, and 1000× solutions were sterilized via membrane filtration using a 0.22-μm polyethersulfone (PES) filter (Sartorius AG, Göttingen, Germany). The cells were harvested via centrifugation at 4500 rpm and washed with hexane and sterilized water for subsequent experiments. Bean oil was used for PHA production, and nitrogen was supplied by the addition of 1 g/L urea. Various concentrations of bean oil, urea solution, and PHA precursors were used according to the purpose of each experiment. Unless otherwise specified, components used in medium were obtained from Sigma-Aldrich (St. Louis, MO, USA).

### 2.4. PHA Analytical Methods

After cultivation, cells were harvested by centrifugation, and the settled pellet was washed twice using 1 mL of hexane followed by 1 mL of distilled water. The washed cells were transferred to a glass vial and lyophilized, after which the dry cell weight was measured. For methanolysis, an equivalent volume of chloroform and 15% (*v*/*v*) H_2_SO_4_ methanol solution were then added to the vial, and the reaction was performed at 100 °C for 2 h. Then, the samples were cooled to room temperature. A total of 1 mL of deionized water was added to the methyl ester solution, followed by vortexing for 5 s. To remove residual water, the chloroform layer was transferred to a microtube containing anhydrous Na_2_SO_4_. The samples were filtered using a 0.22 μm PVDF filter and transferred to a clean borosilicate vial. Next, the prepared samples were analyzed via GC-FID system (Young In Chromass 6500; Seoul, Korea) in split mode (1/10) equipped with a fused silica capillary column (DB-FFAP, 30 mm length, 0.320 mm internal diameter, and 0.25 film; Agilent Technologies, Santa Clara, CA, USA). Helium was used as the carrier gas at a flow rate of 3.0 mL/min. The oven temperature was initially held at 80 °C for 5 min, increased to 220 °C at a rate of 20 °C/min, and then maintained at 220 °C for 5 min. The injector and FID temperature were maintained at 210 °C and 230 °C, respectively.

### 2.5. RT-qPCR Analysis

To compare the transcriptional levels of genes related to fatty acid metabolism, *Cupriavidus* sp. Oh_1 and H16 were cultivated in of 5× ReMM medium containing 3% bean oil (*v*/*v*), 0.1% urea (*w*/*v*), 100× MgSO_4_, 100× CaCl_2_, and 1000× trace elements at 30 °C for 24 h. mRNA was extracted using RNeasy Mini Kits (QIAGEN, Hilden, Germany), following the protocol. SuperScript^TM^ III First-Strand Synthesis System (Invitrogen, Waltham, MA, USA) was used for cDNA synthesis. Real-time PCR was performed on a StepOnePlus (Applied Biosystems^TM^, Foster City, CA, USA) system after normalizing the concentrations of the synthesized cDNA. TOPreal^TM^ qPCR 2X PreMIX (SYBR Green with high ROX; Enzynomics) was used for reaction.

### 2.6. Fed-Batch Fermentation Conditions

Precultures were prepared in two 250 mL flasks, each containing 50 mL of medium, at 30 °C for 24 h. Preculture conditions were identical to those of the main fermentation culture, and resulting preculture fluid was directly inoculated into the fermentor without additional pretreatment. Fed-batch fermentation for PHA production was performed in a 5 L fermentor (CNS, Deajeon, Korea) with a working volume of 2L. The initial medium composition was as follows: 4 g/L NaH_2_PO_4_, 4.6 g/L Na_2_HPO_4_, 0.45 g/L K_2_SO_4_; 0.39 g/L MgSO_4_; 0.062 g/L CaCl_2_; 0.0015 g/L FeSO_4_•7H_2_O, 0.0024 g/L MnSO_4_•H_2_O, 0.0024 g/L ZnSO_4_•7H_2_O, 0.00048 g/L CuSO_4_•5H_2_O, 30 g/L of bean oil, and 10 g/L NH_4_NO_3_. Additional oil was continuously supplied at a rate of 2.3 g/L per h after 12 h of fermentation. Feeding continued until a total 300 g of soybean oil had been added, after which it was stopped. The pH was adjusted to 6.8 using NaOH as the base and phosphoric acid as the acid. The temperature was maintained at 30 °C. To maintain the dissolved oxygen (DO) level at 40%, the agitation speed was initially set at 200 rpm and was increased to 600 rpm during cultivation. After culturing for 183 h, the cells were harvested, utilized for PHA films preparation, and analyzed for PHA production.

### 2.7. Analysis of Physical and Thermal Characteristics

GPC (gel permeation chromatography) was used to measure the number average molecular weight (Mn), weight average molecular weight (Mw), and polydispersity index (PDI) [23]. The analysis was performed using an HPLC system (YOUNGIN Chromass, Seoul, Korea) equipped with a loop injector (Rheodyne 7725i), isocratic pump system (YL9112), column oven (YL9131) with three columns (K-G 4A, guard column; K-804 8.0 × I.D. × 300 mm; K-805, 8.0 × 300 mm; Shodex), and a refractive index detector (YL9170). Chloroform was used as the mobile phase at a 1 mL/min at 40 °C. A 20 μL of each prepared sample was injected, and M_W_ was calculated through a calibration curve based on polystyrene standards ranging from 5000 to 2,000,000 Da.

A universal testing machine (UTM) was used to determine the Young’s modulus (YM), tensile strength (TS), and elongation at the break (EL) of the samples. UTM was performed using a EZ-SX (UTM; Shimadzu, Kyoto, Japan). The samples were cut into dimensions of 10 × 60 mm, and the gauge lengths were set accordingly. The tests were conducted at a crosshead speed of 10 mm/min. Elongation at break (EL) was calculated using Equation (1):

*EL* = (*d_after_* − *d_before_*)∕*d_before_* × 100 (1)

where ***d*** refers to the grips-to-grip distance before and after the samples fractured.

Differential scanning calorimetry (DSC) analysis was performed on extracted PHA films. NEXTA DSC 200 instrument (Hitachi High-Tech Corporation, Tokyo, Japan) was used to analyze the thermal properties of PHA films containing 3HB and 3HHx units [1]. For analysis, approximately 5–6 mg of each sample was sealed in a standard aluminum pan. The measurement was performed under a nitrogen atmosphere. The temperature program included both heating and cooling at a rate of 10 °C/min as follows: 30 °C, 7 min → −60 °C, 10 min → 190 °C, 10 min (first heating) → −60 °C, 2 min → 30 °C, 10 min → −60 °C, 10 min → 190 °C, 10 min (second heating) → −60 °C, 0 min. The crystallization temperature (Tc), melting temperature (Tm), and glass transition temperature (Tg) of the polymers were determined via second heating.

## 3. Results and Discussion

### 3.1. Identification of a High PHB-Producing Strain from Soybean Oil

**Figure 1:**
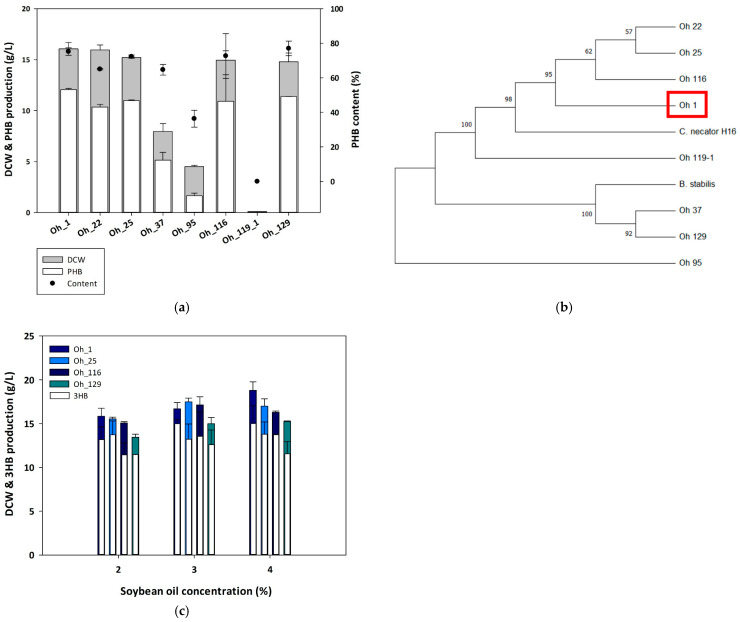
Screening and identification of polyhydroxybutyrate (PHB)-producing strain from soybean oil. (**a**) Comparison of eight strains using GC analysis to identify PHB production. (**b**) Phylogenetic tree based on 16S rRNA sequencing of *Cupriavidus* sp. Oh_1. (**c**) Optimal soybean oil concentration for PHB production in *Cupriavidus* sp. Oh_1, Oh_25, Oh_116, and Oh_129.

PHB-producing strains were isolated from soil and freshwater samples collected in Korea. After dilution with sterile water, the samples were spread onto TSB agar plates. To screen for oil-utilizing strains, isolated colonies were cultured in ReMM supplemented with soybean oil as the sole carbon source to evaluate their capacity for PHB production. Among them, eight strains were identified with the ability to produce PHB using soybean oil as the sole carbon source (Figure 1a). When cultivated in a medium containing 2% soybean oil and 0.1% urea, Oh_1, Oh_25, Oh_116, and Oh_129 produced substantially higher amounts of PHB among the eight identified strains. Oh_1 and Oh_25 exhibited comparable PHB yields, ranging from 10.36 to 12.05 g/L, with a DCW of 15.95–16.05 g/L. Oh_116 and Oh_129 also exhibited similar PHB production ranging from 10.92 to 11.38 g/L of PHB, with a 14.8–14.95 g/L of biomass.

Strain identification was conducted based on the 16S rRNA sequences analysis of the eight strains and a phylogenetic tree was constructed (Figure 1b). The 16S rRNA sequences of *C. necator* H16 (E6A55_RS08365) and *Bacillus stabilis* (BSTAB16_RS27040) were used as the reference sequences. The analysis revealed that five of the isolated strains belonged to *Cupriavidus* sp., while the other three strains were identified as *B. stabilis*. Notably, Oh_1, Oh_25, and Oh_116, which exhibited relatively high DCW and PHB production from soybean oil, showed high similarity to *C. necator* H16, which is known for its ability to produce large amounts of PHB from plant oil as a carbon source [24].

To identify strains that produced the highest amounts of PHB from soybean oil, PHB production was analyzed at various soybean oil concentrations ranging from 2 to 4% using the four strains that exhibited the highest PHB yields (Figure 1c). The results showed that at 2% of soybean oil, Oh_25 produced 13.73 g/L of PHB with a DCW of 15.6 g/L. However, at 3% of soybean oil, Oh_1 had the highest PHB yield, producing 15.23 g/L of PHB with a DCW of 17.2 g/L.

Although Oh_25 exhibited higher PHB production at lower soybean oil concentrations, Oh_1 achieved the highest overall PHB yield when more carbon sources were available. As maximizing PHA production is crucial for industrial applications, Oh_1 was selected for the further characterization and examination of its PHA production potential, and 3% soybean oil was selected as the optimal concentration for the subsequent experiments.

### 3.2. Optimization of Culture Conditions for PHB Production

**Figure 2:**
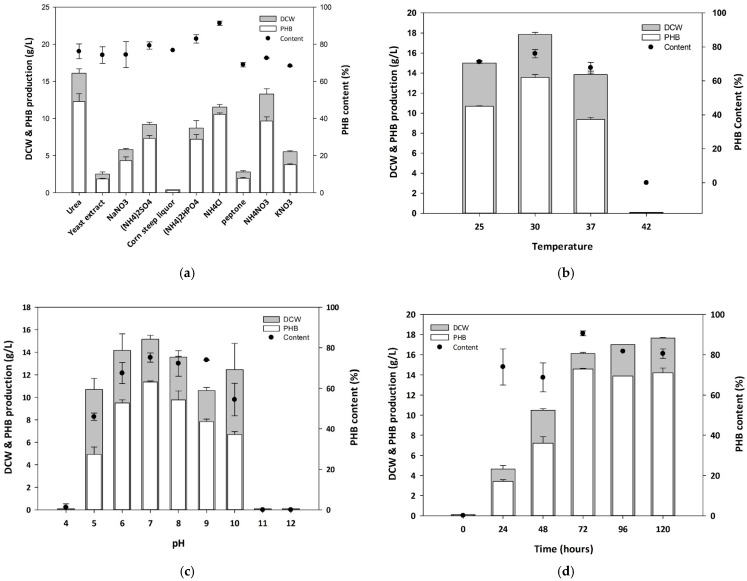
Optimization of culture conditions for PHB production. Effect of (**a**) nitrogen source, (**b**) temperature, and (**c**) pH on PHB production using 5× ReMM medium with 3% soybean oil. (**d**) Time-dependent monitoring of PHB production.

Nitrogen was an essential nutrient for the growth and metabolic activity of bacterial strains [25]. Microbial growth and PHB production were affected by the types of carbon and nitrogen sources and their relative ratio (C/N) [26]. To determine the most appropriate nitrogen source for maximizing PHB production, 0.1% of 10 different nitrogen sources were added to 5× ReMM medium containing 3% of soybean oil. After 72 h, Oh_1 exhibited the highest PHB production when urea was used as the nitrogen source (Figure 2a).

Culture temperature was then optimized. Depending on the temperature, the microbial growth, enzyme activity, and PHB production yield could change. High temperatures decreased PHA synthase activity and stimulated protein denaturation [27]. To optimize the temperature at which protein activity was most active to maximize PHB production, Oh_1 was cultivated in 5× ReMM medium with 3% of soybean oil at 25, 30, 37, and 42 °C. The PHB production was the highest when cultured at 30 °C (Figure 2b).

In addition to nitrogen and temperature, pH was a significant parameter for metabolic activity, cell growth, and PHB accumulation. Various PHA synthases showed the highest activity at their optimal pH. However, beyond that range, enzyme activity decreased, which could lead to lower PHB productivity [28,29]. To determine the optimal pH for PHB production, Oh_1 cell was cultured under various pH conditions. The highest cell growth, PHB production, and PHB content were observed at pH 7 (Figurer 2c).

In the optimized medium, the growth of *Cupriavidus* sp. Oh_1 was assessed every 24 h for 5 days. The highest PHB production was observed after 72 h of cultivation, producing 14.57 g/L of PHB with a DCW of 16.1 g/L (Figure 2d). After 72 h, PHB production decreased slightly owing to the depletion of available nutrients necessary for cell growth [30,31].

### 3.3. Copolymer Synthesis via Supplementation with Various Monomers and the Comparison of P(3HB-co-3HHx) Production of Cupriavidus sp. Oh_1 and H16

PHB was the most common type of PHA. However, its high crystallinity and melting point limited its industrial applications. To overcome these challenges, many studies have focused on synthesizing copolymers by combining P(3HB) with other monomers, such as 3HP, 3HV, 4HV, or 5HV. These copolymers enhanced the physical and chemical properties of PHB, making their synthesis essential [32,33].

To identify the ability of *Cupriavidus* sp. Oh_1 to synthesize various monomers, 0.1% of 3-hydroxypropionic acid (3-HPA), 4-hydroxybutyric acid (4-HBA), 4-hydroxyvaleric acid (4-HVA), and 5-hydroxyvaleric acid (5-HVA) were supplied (Table 2). Oh_1 was unable to synthesize 3HP; however, 4HB, 3HV, 4HV, and 5HV were incorporated into the PHA, with 3HV showing a molar fraction of approximately 1%. Notably, 3HV is synthesized when 4HVA was supplied as a precursor, and its molar fraction could be affected by the substrate specificity of the PHA synthase [34,35]. Although 3HV was synthesized at a higher molar fraction than the other monomers and showed a slightly higher molar fraction than *C. necator* H16, it remained too low to significantly improve the physical properties of PHB.

### 3.4. Time-Dependent Monitoring of P(3HB-co-3HHx) Production by Cupriavidus sp. Oh_1

3HHx, a six-carbon medium-chain-length polyhydroxyalkanoate monomer, exhibited high elasticity, low crystallinity, and high elongation at break. P(3HB-*co*-HHx) with 17 mol% 3HHx has mechanical and thermal properties comparable to those of low-density polyethylene (LPDE) and demonstrated superior processing abilities compared to PHA homopolymers [36]. P(3HB-*co*-3HHx) could be produced from plant oil by introducing the *phaC* gene, which facilitated 3HHx incorporation, and the *phaJ* gene, which supplied the 3HHx-CoA monomer for copolymer synthesis [37].

**Figure 3:**
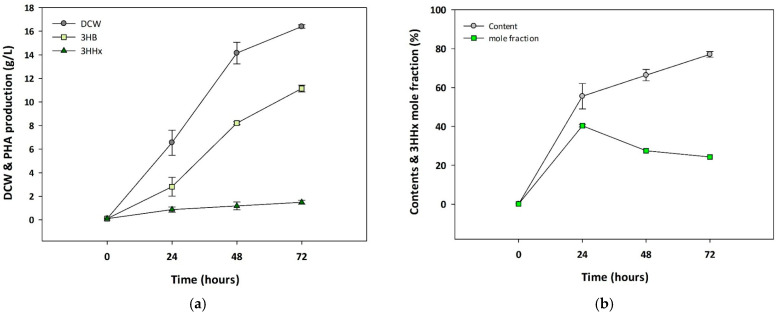
Time-dependent monitoring of P(3HB-*co*-3HHx) production in *Cupriavidus* sp. Oh_1. (**a**) Analysis of DCW and PHA production. (**b**) Analysis of the PHB content and 3HHx mole fractions.

To investigate whether *Cupriavidus* sp. Oh_1 could produce P(3HB-*co*-3HHx) using soybean oil as the sole carbon source, a vector harboring *phaC* from *R. aetherivorans* I24 and *phaJ* from *P. aeruginosa* was introduced into the strain. The strain *Cupriavidus* sp. Oh_1/*phaCJ* was cultivated in 3% of soybean oil, and PHA production was monitored at different time points. The synthesis of 3HHx was first detected at 24 h, and after 72 h, 1.49 g/L 3HHx was produced with a DCW of 16.4 g/L. The mole fraction of 3HHx reached a specific level of 24.3% (Figure 3). These results demonstrated that the overexpression of *phaC_Ra_* enabled the incorporation of 3-hydroxyhexanoly-CoA into P(3HB-*co*-3HHx) production, and *phaJ_Pa_* enhanced the synthesis of the 3-hydroxyhexanoyl-CoA precursor, resulting in 3HHx accumulation in P(3HB-*co*-3HHx) without the need for the genetic modification of PHA biosynthesis-related genes.

**Figure 4:**
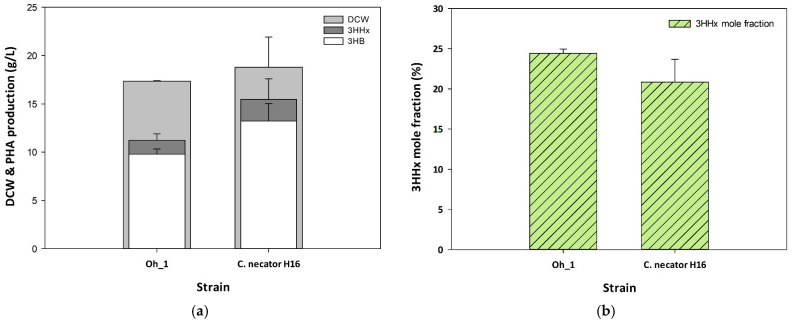
Comparison of *Cupriavidus* sp. Oh_1 and H16 P(3HB-*co*-3HHx) production by (**a**) DCW, PHA production, and (**b**) 3HHx mole fraction.

To evaluate P(3HB-*co*-3HHx) production in *Cupriavidus* sp. Oh_1, an equivalent *phaC_Pa_J_Ra_* vector was introduced, and the production levels in *Cupriavidus* sp. Oh_1 and H16 were analyzed. Both strains were cultured under optimized conditions for 72 h. The amount of PHB produced by Oh_1/*phaCJ* was lower than that of *C. necator* H16/phaCJ at 10.59 g/L and 17.22 g/L, respectively (Figure 4a). However, the 3HHx mole fraction accumulated by Oh_1/*phaCJ* was significantly higher than that of *C. necator* H16/*phaCJ*, reaching 24.4% and 20.8%, respectively (Figure 4b). These results suggest that *Cupriavidus* sp. Oh_1 exhibits a stronger tendency to incorporate 3HHx into the copolymer despite its lower overall polymer production.

### 3.5. Fed-Batch Fermentation Using Cupriavidus sp. Oh_1/phaCJ

**Figure 5:**
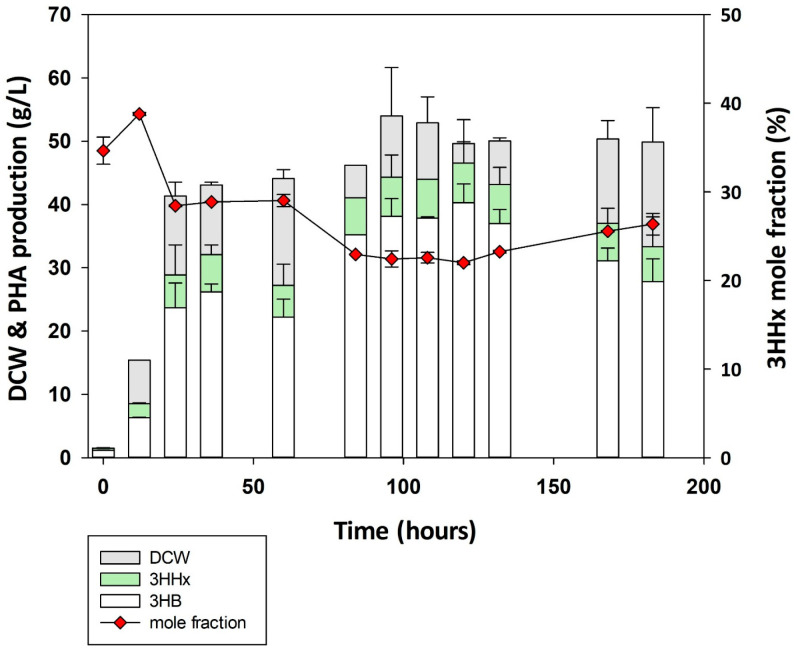
Poly(3HB-*co*-3HHx) production by fed-batch fermentation from soybean oil in Oh_1/*phaC_Ra_phaJ_Pa_*.

To produce P(3HB-*co*-3HHx) on a large scale from soybean oil, fermentation was performed in a 5 L jar fermentor for 183 h using *Cupriavidus* sp. Oh_1/*phaC*_Ra_*J*_Pa_ (Figure 5). Based on previous optimization experiments, 3% soybean oil and 0.1% of NH_4_NO_3_ were used as the initial carbon and nitrogen sources, respectively, and 300 g/L of soybean oil was supplied for 5 days. Oh_1 started accumulating PHB after 12 h, and both DCW and PHB levels continued to increase until 120 h; at this point, Oh_1 showed the highest PHA production of approximately 48.93 g/L in 52.3 g/L of DCW. At the end of the fermentation, Oh_1 accumulated 37.01 g/L of PHA in 50.35 g/L of DCW, with a 3HHx mole fraction of 25.56%. After 120 h, a gradual decrease in DCW and PHA due to nutrient depletion was observed. These results suggest that Oh_1 is a promising strain for sustainable PHA production with high production yield, stable growth, and a high 3HHx mole fraction, maintaining a consistent copolymer composition while effectively utilizing soybean oil for practical applications.

### 3.6. Copolymer of the Physical Properties of PHB and PHBHHx Produced by Cupriavidus sp. Oh_1 and H16

PHB and PHBHHx with different molar fractions of 3HHx produced from soybean oil by *Cupriavidus* sp. Oh_1 were extracted and their physicochemical properties were analyzed using UTM, DSC, and GPC (Table 3). By comparing the mechanical properties of the PHB and PHBHHx films using UTM, the elongation at break significantly increased for the PHBHHx film produced by *Cupriavidus* sp. Oh_1/*phaCJ* and H16/*phaCJ* compared with that of PHB, which exhibited brittle and stiff characteristics. The elongation at break of the PHBHHx film with a 3HHx mole fraction of 27.2% produced by *Cupriavidus* sp. Oh_1/*phaCJ* was 179.7 ± 10.2%, which was higher than that of the film produced by H16/*phaCJ* with a 22.5% mol% of 3HHx. In contrast, the tensile strength and Young’s modulus decreased significantly by 3.6 ± 0.1 MPa and 22.2 ± 3.5 MPa, respectively. This is because, as the 3HHx integration ratio in the PHA structure increases, the flexibility of the copolymer improves, while its crystallinity decreases, making it more ductile [38].

In the DSC analysis, the Tm and ΔH of the PHB film from wild type Oh_1 were 170.9 °C and 28.6 J/g, respectively, while Tg and Tc were not detected. The ΔH of P(3HB-*co*-3HHx) film from Oh_1/*phaCJ* and H16/*phaCJ* was lower than that of the PHB film. Notably, the P(3HB-*co*-27.2 mol% 3HHx) copolymer from Oh_1/*phaCJ*, exhibited reductions in Tg and Tc, decreasing to −6.29 °C and 58.77 °C, respectively. Additionally, a lower ΔH (16.3 J/g) was identified compared to the copolymer produced by *C. necator* H16/*phaCJ* (16.6 J/g). This reduction in crystallinity and thermal properties may contribute to the enhanced flexibility and processability of the copolymer films compared with those of PHB.

For GPC analysis, the P(3HB-*co*-3HHx) film from Oh_1/*phaCJ* had a molecular weight (Mw) of 12.3 × 10^5^ and a polydispersity index (PDI) of 1.41. Although the Mw of the P(3HB-*co*-3HHx) film from Oh_1/*phaCJ* was lower than that of the PHB film produced by wild-type Oh_1, it was significantly higher than that of the P(3HB-*co*-3HHx) film synthesized using Re2133/pCB81, a strain in which *phaB* and *phaC* were deleted. Previous studies have shown that genome-deleted strains typically produce PHA with reduced molecular weights, such as a Mw of 14.6 × 10^4^ and a Mn of 25.7 × 10^4^, even when achieving a high 3HHx mole fraction. In the case of Re2133/pCB81, the reduction in 3HB-CoA availability resulting from *phaB* deletion led to lower Mw and Mn values [10,14,38]. While the reduction in Mw and Mn could be attributed to various complicated factors, the Oh_1/*phaCJ* strain, without any gene deletions, may contribute to advancing PHBHHx biosynthesis with improved 3HHx content and high molecular weight characteristics.

### 3.7. mRNA Expression Analysis of Fatty Acid β-Oxidation-Encoding Genes in Cupriavidus sp. Oh_1

To identify the reason for the higher 3HHx mole fraction in *Cupriavidus* sp. Oh_1, the expression levels of key genes involved in the *β*-oxidation pathway, including *fadE* (acyl-CoA dehydrogenase), *fadB* (enoly-CoA hydratase), *fadA* (3-ketoacyl-CoA thiolase), *had* (3-hydroxy-CoA dehydrogenase), *phaC*_Ra_, and *phaJ*_Pa_, were analyzed. Oh_1/*phaCJ* cells were cultured under optimized conditions for 24 h, after which mRNA was extracted, and RT-qPCR was performed. The *rpoD* gene was used as a reference gene for normalization, as it is a well-known housekeeping gene in various Gram-negative bacteria [39,40,41]. The *C. necator* H16 strain was used as the control.

**Figure 6:**
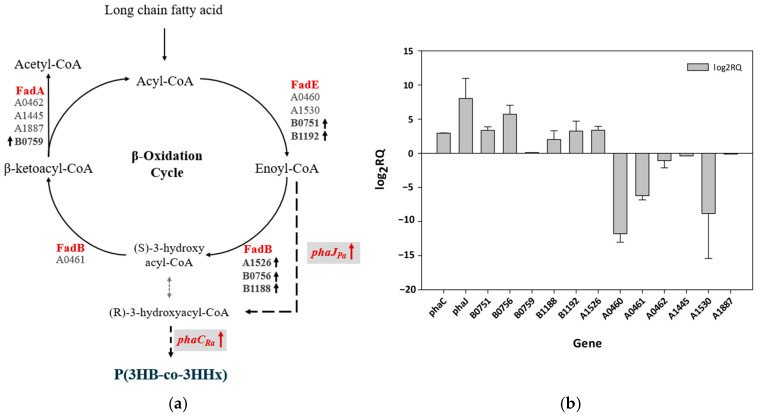
Comparison of β-oxidation-related gene expression Via RT-qPCR of *Cupriavidus* sp. Oh_1 and H16. (**a**) Metabolic pathway of β-oxidation cycle. (**b**) Gene expression variation related to the β-oxidation cycle in *Cupriavidus* sp. Oh_1.

In the initial step of the *β*-oxidation cycle, introduced FAs were activated by acyl-CoA ligase, forming acyl-CoA, which was subsequently oxidized to *trans*-Δ^2^-enoyl-CoA by acyl-CoA dehydrogenase. Enoyl-CoA hydratase then catalyzed the hydration of *trans*-Δ^2^-enoyl-CoA to produce 3-hydroxyacyl-CoA, which was further oxidized to β-ketoacyl-CoA by 3-hydroxy-CoA dehydrogenase. Finally, 3-ketoacyl-CoA thiolase cleaved an acetyl-CoA molecule from β-ketoacyl-CoA [42]. Additionally, *phaJ* catalyzed the hydration of *trans*-Δ^2^-enoyl-CoA to (R)-3-hydroxyacyl-CoA, increasing the available monomers for incorporation into P(3HB-*co*-3HHx), and *phaC* catalyzed the incorporation of (R)-3-hydroxyacyl-CoA into copolymers [4]. Various *β*-oxidation enzyme-encoding gene clusters existed, and several genes present in both *C. neactor* H16 and Oh_1 were selected for analysis through PCR amplification (Figure 6a). Gene expression levels were compared under optimized culture conditions. The gene operons B0751, B0756, B1188, and B1192 in Oh_1/*phaCJ* exhibited log2 fold changes in expression of up to 3.5, 5.72, 2.02, and 3.25, respectively. Notably, *phaC*_Ra_ and *phaJ*_Pa_ showed significantly higher expression levels than *C. neactor* H16/*phaCJ*, with log2 fold changes of 2.94 and 8.2, respectively (Figure 6b).

After introducing *phaC*_Ra_ and *phaJ*_Pa_ into Oh_1, the expression levels of *fadB* were analyzed and compared with those of wild type Oh_1 (Supplementary Figure S1). The results showed that the log2 fold changes of all gene clusters were downregulated. A1526 was a *fadB* homolog, and its functional loss could increase the incorporation of a net amount of 3HHx monomers into the copolymer, thereby increasing the fraction of 3HHx [11]. Therefore, in Oh_1, the introduction of *phaCJ* reduced the expression of A1526 with other *fadB* gene clusters, enhancing the *phaJ*-mediated pathway from 2-enoyl-CoA to (R)-3HHx-CoA, and promoting the copolymerization of (R)-3HHx-CoA.

Overall, owing to the complex intracellular metabolic processes and numerous unidentified genes in the novel strain Oh_1, it was challenging to definitively determine whether specific genes were upregulated or downregulated as a direct cause of variations in the 3HHx mole fraction. However, based on these results here, Oh_1 exhibited high activity in the B0700 and B1100 operons, along with increased activity of the introduced *phaC*_Ra_ and *phaJ*_Pa_. This suggests that these factors enhanced the supply of monomers for P(3HB-*co*-3HHx) synthesis, ultimately leading to a higher 3HHx molar fraction.

## 4. Conclusions

Given the promising future of [P(3HB-*co*-3HHx)] copolymers as advanced PHAs for practical applications, owing to their superior flexibility and similarity to various petroleum-based polymers [3] compared to P(3HB) homopolymers, the ability to control the 3HHx fraction was crucial for producing a range of [P(3HB-*co*-3HHx)] materials with varying properties. Historically, harsh genetic engineering approaches, such as the deletion of *phaB* and *phaC* to block P(3HB) synthesis, have been considered the most effective strategies to increase the 3HHx mole faction [10,14]. In contrast, our approach offered a simpler alternative by identifying a novel strain capable of high 3HHx production. *Cupriavidus* sp. Oh_1 proficiently metabolized various concentrations of soybean oil and urea, leading to increased PHA accumulation. Notably, Oh_1 achieved a 27.2 mol% 3HHx fraction using soybean oil as the sole carbon source, enabled solely by the introduction of *phaC*_Ra_ and *phaJ*_Pa_. To identify the reasons for high 3HHx incorporation, the expression levels of various operons involved in the *β*-oxidation pathway, which was the crucial fatty acid metabolism route, were analyzed. Oh_1 exhibited the higher expression of specific operons and increased affinity for *phaC*_Ra_ and *phaJ*_Pa_. Additionally, Oh_1 produced more EPS, which may act as a natural detergent to aid oil utilization (Supplementary Figure S2), although EPS production can sometimes hinder recovery processes.

While the optimal 3HHx content might vary depending on the industrial application, increasing the 3HHx fraction remains valuable, even though a higher content does not always directly translate to better copolymer properties. Importantly, the identification of a strain inherently capable of high 3HHx synthesis without requiring genome deletions was a significant advancement. Continued efforts to discover novel strains will further expand strategies for controlling 3HHx incorporation. Although the observed results could partly stem from the heterologous *phaC*, the native *phaC* of Oh_1 might possess unexplored potential for synthesizing various copolymers and remain a promising candidate for further study. Further investigations of this metabolic pathway may provide novel strategies for enhancing PHA biosynthesis.

## Figures and Tables

**Table 2:** Evaluation of various monomer synthesis by *Cupriavidus* sp. Oh_1 and H16.

Strain	Precursor (0.1%)	DCW (g/L)	PHA (g/L)	PHA Mole Fraction (%)					
				3HB	3HP	4HB	3HV	4HV	5HV
Oh_1	3HP	15.2	14.3	99.7	-	-	0.3	-	-
	4HB	16.3	15.4	99.2	-	0.5	0.3	-	-
	4HV	14.95	13.7	98.5	-	-	1.3	0.2	-
	5HV	16.9	15.1	99.2	-	-	0.4	-	0.4
*C. necator* H16	3HP	21.5	17.8	99.8	-	-	0.2	-	-
	4HB	19	16.99	99.4	-	0.3	0.2	-	-
	4HV	18.5	16.51	98.8	-	-	1.2	0.1	-
	5HV	22.3	20.45	99.5	-	-	0.3	-	0.2

**Abbreviations**: DCW, dry cell weight; PHA, polyhydroxyalkanoate; HV, hydroxyvalerate; HB, hydroxybutyrate; HP, hydroxypropionate.

**Table 3:** Thermal and physical properties of PHB and PHBHHx synthesized using soybean oil.

	3HHx (mol%)	UTM			DSC				GPC		
		TS (MPa)	EL (%)	YM (MPa)	Tg (°C)	Tc (°C)	Tm (°C)	ΔH (J/g)	Mn (10^5^)	Mw (10^5^)	PDI
Oh_1 wt	0	9.9 ± 5.9	11.7 ± 5.2	488.6 ± 122.6	n.a.	n.a.	170.9	28.6	10.9	17.4	1.66
Oh_1/*phaCJ*	27.2	3.6 ± 0.1	179.7 ± 10.2	22.2 ± 3.5	−6.29 ± 3.0	58.77	176.1	16.3	8.7	12.3	1.41
H16/*phaCJ*	22.5	5.8 ± 0.3	152.3 ± 6.2	1225 ± 172	−0.37	71.61 ± 0.2	174.2	16.6	6.7	14.4	2.16

**Abbreviations**: HHx, hydroxyhexanoate; UTM, universal testing machine; TS, tensile strength; EL, elongation at break; YM, Young’s modulus; DSC, differential scanning calorimetry; Tg, glass transition temperature; Tc, crystallization temperature; Tm, melting temperature; GPC, gel permeation chromatography; Mn, number average molecular weight; Mw, molecular weight; PDI, polydispersity index.

## Data Availability

All data related to the study are provided in the manuscript and its associated files.

## Supplementary Materials

The following supporting information can be downloaded at https://www.mdpi.com/article/10.3390/polym17131824/s1. Figure S1: Log2 fold changes in *fadB* gene clusters encoding *β*-oxidation enzymes in *Cupriavidus* sp. Oh_1/*phaCJ* strain with Oh_1 wild type; Figure S2: Comparison of EPS production of *Cupriavidus* sp. Oh_1 and H16 in 5x ReMM medium with 3% soybean oil and 0.1% of urea.

## Abbreviations

The following abbreviations are used in this manuscript:

DSC	Differential scanning calorimetry
GPC	Gel permeation chromatography
MCL	Medium-chain length
ReMM	*Ralstonia eutropha* minimal media
SCL	Short-chain length
TSA	Tryptic soy agar
TSB	Tryptic soy broth
