# Supplementary figures and images for: Discovery of a High 3-Hydroxyhexanoate Containing Poly-3-hydroxybutyrate-co-3-hydroxyhexanoate Producer-, Cupriavidus sp. Oh_1 with Enhanced Fatty Acid Metabolism
Source: Polymers (Basel). 2025 Jun 30;17(13):1824. doi: 10.3390/polym17131824 (PMC12251744; doi:10.3390/polym17131824)

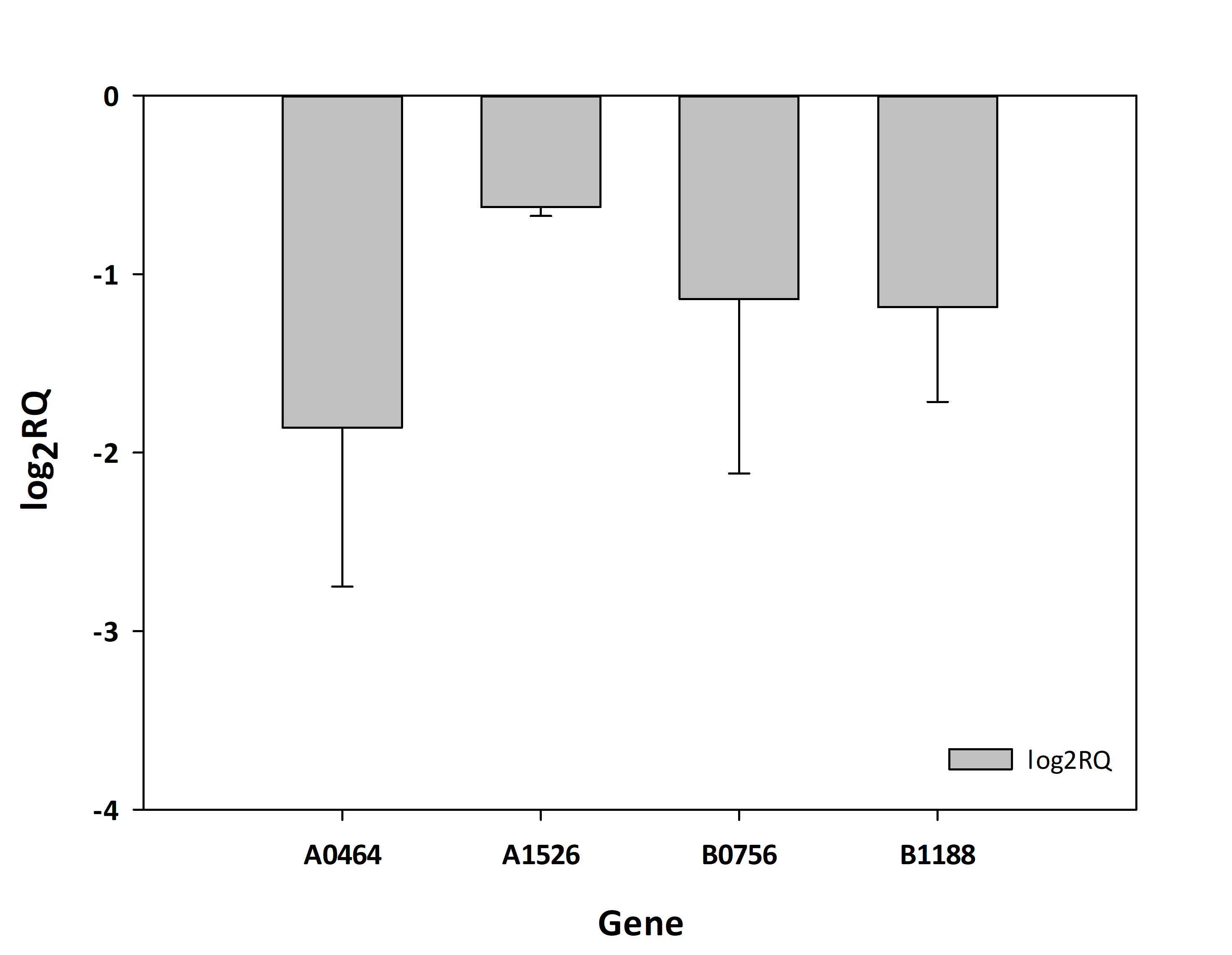

Supplement: Supplementary file 1 [file polymers-17-01824-s001.zip › SUPPLE/Figure S1_FadB_RT-qPCR.JPG]

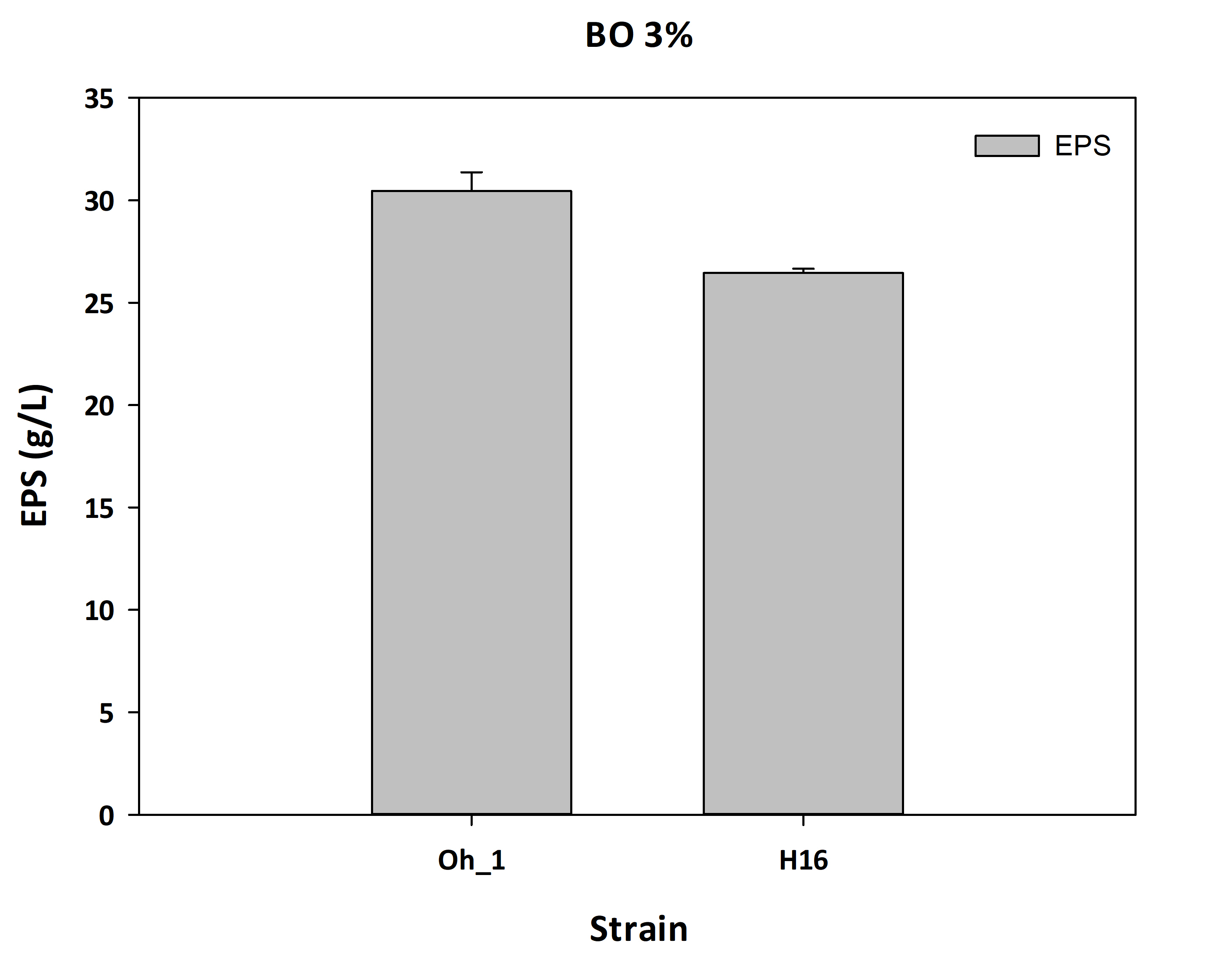

Supplement: Supplementary file 1 [file polymers-17-01824-s001.zip › SUPPLE/Figure S2_EPS production.JPG]
